# Early Risk Factors for Self-Injurious Thoughts and Behaviours: A UK Population-Based Study of 219,581 People

**DOI:** 10.3390/bs14010016

**Published:** 2023-12-24

**Authors:** Alejandro Porras-Segovia, Ana Pascual-Sanchez, Geva Greenfield, Hanna-Marie Creese, Sonia Saxena, Dougal Hargreaves, Dasha Nicholls

**Affiliations:** 1 Division of Psychiatry, Department of Brain Sciences, Imperial College London, London W12 7T, UK; 2 Health Research Institute Fundación Jiménez Díaz, 28040 Madrid, Spain; 3 Department of Psychiatry, University Hospital Rey Juan Carlos, 28933 Madrid, Spain; 4 CAMHS Enhanced Treatment Service, South London and Maudsley NHS Foundation Trust, London SE5 8AZ, UK; 5 Department of Primary Care and Public Health, Imperial College London, London SW7 2AZ, UKh.creese@imperial.ac.uk (H.-M.C.);; 6 Department of Epidemiology and Biostatistics, School of Public Health, Imperial College London, London W2 1PG, UK

**Keywords:** suicide, suicide ideation, suicide attempt, non-suicidal self-injury, child and adolescent mental health

## Abstract

Mental disorders are a major problem among young people. To identify early risk factors of self-injurious thoughts and behaviours (SITB) among young adults with mental health problems, this case–control study drew data from the Clinical Practice Research Datalink (CPRD), a primary care database covering 8% of the UK population. We explored the role of early factors (presenting at 8–14 years old) for suicidal ideation, suicide attempts, and non-suicidal self-injury (NSSI) in young adulthood (age 18–25 years) by performing logistic regressions. Our sample consisted of 219,581 participants, of which 6.51% had at least one SITB in young adulthood. Early risk factors for SITB included early NSSI, suicidal ideation, sexual abuse, behavioural problems, and mood and psychotic symptoms. Frequency of GP visits had a protective effect. Lack of access to mortality data, ethnicity, and socioeconomic status was a limitation of the current study. In conclusion, early symptoms in late childhood/early adolescence can be the start of long-standing problems going into adult life. The training of primary care providers in suicide risk assessment and proper co-ordination with child and adolescent mental health services are crucial for suicide prevention.

## 1. Introduction

Suicide is a major public health problem worldwide, representing the fourth leading cause of death among people aged 15–29 [1]. In the United Kingdom (UK), self-inflicted injuries were the cause of 25.2% of deaths among people aged 15–24 in 2019 [1]. Since it affects young people, the years of potential life lost (YPLL) due to suicide are very high. For instance, a study showed that, in the United States of America, the YPLL due to suicide in the year 2018 were 1,344,552 YPLL, very close to the 1,591,487 YPLL caused by COVID-19 in the year 2020 [2]. In other areas of the world, the YPLL due to suicide in 2020 were even higher than that attributed to COVID-19 in the same year [3]. 

Among the most prominent risk factors for death by suicide are suicide attempts (suicide attempts), suicidal ideation, and non-suicidal self-injury (NSSI). Suicidal ideation refers to thoughts or contemplation about ending one’s own life. These thoughts can vary from fleeting considerations about death to more established plans and intentions. Although suicidal ideation is one of the most significant risk factors of future death by suicide [4], many people with suicidal ideation will never perform a suicide attempt [5]. Indeed, it has been found that only one third of patients experiencing suicidal ideation will attempt suicide [6]. Nevertheless, suicidal ideation indicates significant emotional distress that requires attention and intervention. 

A suicide attempt refers to an intentional action taken to end one’s own life and which does not have a fatal outcome. Most people who attempt suicide will not die by suicide, as suicide attempts are estimated to be at least 20 times more frequent than deaths by suicide [1]. NSSI is also a relevant phenomenon highly related to suicide. It refers to deliberate and self-inflicted damage to one’s body tissue without intending to end one’s life. NSSI can take various forms, such as cutting, burning, scratching or hitting. NSSI affects particularly the youth population, who may engage in NSSI as a way to manage overwhelming psychological pain as they may feel a temporary sense of relief or control even though it is not a healthy or effective long-term coping mechanism [7]. Although non-suicidal by definition, NSSI increases the risk for suicide attempt [5,8,9] and is associated with other mental health problems [10,11].

Taken together, these behaviours can be referred to as self-injurious thoughts and behaviours (SITB), and they are not only risk factors for death by suicide but also have a significant impact in their own right in terms of healthcare costs, functionality, and quality of life [12,13,14]. We must recognise their relevance beyond the risk of death that they represent. NSSI, suicidal ideation, and suicide attempts share certain characteristics but are ultimately distinct phenomena, each with their own risk factors [4,5]. In the meta-analysis by Franklin et al. [4], it was shown that certain risk factors, such as previous history of suicidality, were equally important for both suicidal ideation and suicide attempts. However, other factors showed a greater specificity. For instance, internalising symptoms have been shown to hold a greater association with suicidal ideation than with suicide attempts. Conversely, physical illness seems to be a stronger risk factor for suicide attempts than for suicidal ideation [4]. 

A literature review explored factors associated with NSSI and suicide attempts. It was found that depression and borderline personality traits were more prevalent among those who presented both behaviours than among those who presented only one of them. In contrast, factors that were more associated with NSSI were hopelessness, female gender, and anxiety, while factors that showed more specificity for suicide attempts were suicidal ideation, poor family functioning, and conduct problems, among others. They also found that history of NSSI was an independent factor for engaging in either of these two behaviours [15]. 

Identifying risk factors of these types of behaviour in the young adult population is one of the most relevant fronts for action in suicide prevention [16]. Since about 50% of lifetime mental health problems begin before mid-adolescence [17], early identification of risk factors in childhood and adolescence could contribute to the prevention of SITB. In general, there is a paucity of studies exploring early risk factors for suicidality, and they usually focus on a limited range of risk factors, while not so much attention is paid to others. Although the role played by some early risk factors, such as childhood sexual abuse, in the development of suicidal behaviour is well studied [18], little is known about other potential early risk factors, such as psychotic symptoms or NSSI in childhood. 

For instance, a notable study followed up a community sample of children and their families through several years and found that childhood maltreatment was associated with risk for suicidal attempts in early adulthood. The authors also explored the role of early depression and anxiety, but no exploration of other psychiatric symptoms, including psychosis and behavioural problems, was made [19]. Another study also found that sexual abuse was strongly associated with later risk of suicide, and it explored some mental health problems, including depression, anxiety, conduct disorders, and drug abuse, but psychotic symptoms were not explored and neither was the presence of early SITB, such as presenting with suicidal ideation or NSSI in childhood [20]. 

In addition, and with some notable exceptions, most previous studies do not usually cover long periods of time; the median follow-up period of studies exploring suicide risk factors is 72 months [4]. More research is needed to shed light on understudied risk factors and the relationship between childhood problems and the onset of SITB in adulthood.

Primary care electronic health records may hold relevant information to identify early predictors of SITB and their exploration may provide valuable insight for suicide prevention. Primary care providers represent an important point of access for mental healthcare of children and adolescents. Studies show that 5% of children and adolescents have consultations with their primary care provider due to emotional symptoms [21]. They are usually the first point of contact in healthcare, are responsible for initial identification and treatment, and refer to mental health specialists. Hence, primary care providers are crucial gatekeepers for suicide prevention. Among young people who died by suicide, 10–36% had contacted their primary care provider in the month prior to their death for whatever reason, while 42–82% had contacted them in the year before death [22]. 

The aim of this study was to identify early risk factors of NSSI, suicidal ideation, and suicide attempts among young adults with mental health problems using a primary care database, thus generating a large sample size and a follow-up period of several years.

## 2. Materials and Methods

### 2.1. Setting and Design

This was a case–control study nested in a population-based cohort of people with mental health problems treated in primary care settings in the UK. Our data came from the Clinical Practice Research Datalink (CPRD), a primary care database covering 8% of the UK population [23,24]. CPRD offers the possibility of accessing a large amount of coded information from primary care services, generating a large sample size and a follow-up period of several years. The data in CPRD are pseudo-anonymised and include sociodemographic characteristics, General Practitioner (GP) consultations, clinical variables, and referrals to secondary care units. Data collected in CPRD date back to January/1987 and continue to this day. For our study, data were extracted for the period January/1987 to March/2021. This study was approved by the Independent Scientific Advisory Committee (ISAC) of the Medicines & Healthcare products Regulatory Agency, ISAC protocol number 20_000097, and it complies with the Declaration of Helsinki [25].

All data were collected/recorded by GPs. 

The independent variables were present at ages 8–14, and they consisted of mental-health-related main reasons for consultation, i.e., symptoms coded using the so-called “Read codes”, which are standardised categories. To facilitate the analysis and increase statistical power, refinement work was carried out by merging codes considered to be equivalent and grouping together different but related symptoms. Thus, for instance, “Advice to caretaker regarding child’s behaviour” and “Aggressive behaviour” were grouped into the category “Behavioural problems”, while “Hallucinations” and “Delusions” were grouped into the category of “Psychotic symptoms”. All the variables considered were present from ages 8 to 14. In the case of “History of sexual abuse”, the abuse could have taken place at any age prior to 14 years old.

Our independent variables were:

- Gender;
- Frequency of GP visits;
- Behavioural problems;
- Eating problems;
- Anxiety symptoms;
- Mood symptoms;
- Psychotic symptoms;
- NSSI;
- Suicidal ideation;
- History of sexual abuse.

Our dependent variables (outcomes) were three different SITB: suicidal ideation, suicide attempts, and NSSI. For each of these outcomes, cases were those who presented the outcome at 18–24 years old, while controls were the people who did not. 

### 2.2. Sample

Our sample consists of 219,581 persons born between 1979 and 2003 whose data were included in the CPRD database and who met the following inclusion criteria:

- At age 18–24, participants had any type of mental health problem, as evidenced by at least one of the following:
  ○ Diagnosis of any mental disorder;
  ○ SITB;
  ○ Referral to adult mental health service;
  ○ Repeated prescription of psychotropic medication (antidepressants/antipsychotics/mood stabilizers/stimulants/benzodiazepines).
- All patients must have had at least one consultation between the age of 8 and 14 with their GP, registered in the CPRD.

**Figure 1:**
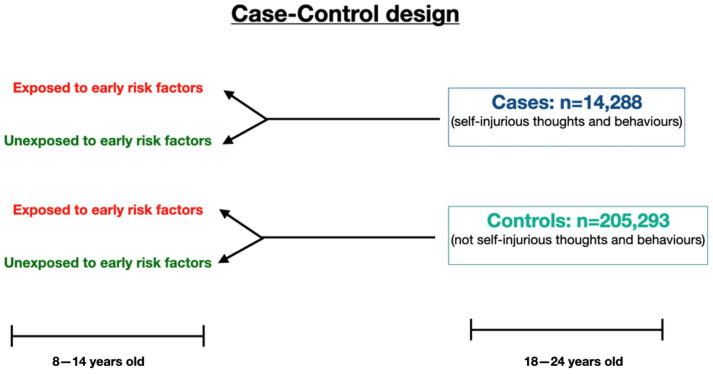
Case–control study design. Both cases and controls had evidence of mental health problems; only cases showed SITB behaviours.

Figure 1 illustrates the study design.

### 2.3. Statistical Analyses

All analyses were performed with Statistical software for data science version 16 (STATA 16). Descriptive data on the sociodemographic and clinical characteristics of the sample are provided. To analyse the early risk factors associated with suicidal ideation, suicide attempt, and NSSI at the age of 18–24 years, logistic regressions were performed. Analyses were controlled for clustering by GP practice, using logistic regression generalised estimating equations (GEE). All tests were two-tailed. Confidence intervals were set at 95% and statistical significance was set at the *p* < 0.05 level.

## 3. Results

### 3.1. Description of the Sample at Age 18–24

Our sample consisted of 219,581 patients with evidence of mental health problems (as defined above) between the ages of 18 and 24, who had received care from their General Practitioner between the ages of 8 and 14. Gender distribution was 62.88% females and 37.11% males. 

Between the ages of 18 and 24 years, 14,288 people (6.51%) had at least some kind of SITB (i.e., at least one GP visit due to NSSI, suicidal ideation, or suicide attempts). Of these, 10.14% had had GP visits for two of the three SITB as young adults, while 0.49% had had visits due to all three outcomes. Among people who attempted suicide, 15.67% had at least one additional GP visit due to suicidal ideation, while 10.95% had a visit due to NSSI. The frequency of the different suicide-related outcomes at age 18–24 years is shown in Table 1. 

### 3.2. Description of the Sample at Age 8–14

Of the 219,581 participants, N = 1034 (0.47%) had at least one GP visit due to NSSI between the ages of 8 and 14, while 297 (0.14%) had at least one GP visit due to SI. No GP visits due to suicide attempts were reported between the ages of 8 and 14. Eight per cent of the sample had at least one potential risk factor, the most common of which was a GP visit due to behavioural problems (5.63% of the sample), followed by a GP visit due to mood symptoms (1.20% of the sample). The mean age of participants at first primary care consultation was 9.67 years (SD = 1.88).

Full baseline characteristics of the sample are shown in Table 2. 

### 3.3. Early Factors Associated with NSSI

Among our sample, 2.83% attended their GP due to NSSI at age 18–24. Seven risk factors between age 8 and 14 were found to be statistically significantly associated with NSSI in young adulthood (age 18–24 years) in the logistic regression. Six of them were identified as risk factors—early NSSI (OR = 3.957, 95% CI: 3.244–4.826), early suicidal ideation (OR = 2.455, 95% CI: 1.657–3.636), history of sexual abuse (OR = 3.336, 95% CI: 2.059–5.407), behavioural problems (OR = 1.807, 95% CI: 1.651–1.978), mood symptoms (OR = 1.341, 95% CI: 1.102–1.632), and psychotic symptoms (OR = 1.846, 95% CI: 1.170–2.911). Among the protective factors, frequency of GP visits had a small effect (OR = 0.998, 95% CI: 0.997–0.999).

### 3.4. Early Factors Associated with Suicidal Ideation

The proportion of primary care visits due to suicidal ideation at age 18–24 was 3.42%. Early risk factors for suicidal ideation in young adulthood were early NSSI (OR = 2.337, 95% CI: 1.849–2.952), early suicidal ideation (OR = 2.038, 95% CI: 1.346–3.080), history of sexual abuse (OR = 1.870, 95% CI: 1.030–3.388), behavioural problems (OR = 1.570, 95% CI: 1.442–1.705), mood symptoms (OR = 1.370, 95% CI: 1.052–1.533), and psychotic symptoms (OR = 1.905, 95% CI: 1.240–2.926). In contrast, protective factors were female gender (OR = 0.689, 95% CI: 0.655–0.723) and older age at first visit—used as a continuous variable (OR = 0.971, 95% CI: 0.960–0.985). 

### 3.5. Early Factors Associated with Suicide Attempts

The frequency of primary care visits due to suicide attempts was 0.89% (i.e., 0.89% of participants had at least one visit due to a suicide attempt). Early risk factors for suicide attempts in young adulthood were early NSSI (OR = 2.686, 95% CI: 1.770–4.074), history of sexual abuse (OR = 3.958, 95% CI: 1.766–8.872), behavioural problems (OR = 1.586, 95% CI: 1.360–1.849), and older age at first visit (OR = 1.039, 95% CI: 1.014–1.065). Protective factors were female gender (OR = 0.502, 95% CI: 0.458–0.550) and number of GP visits (OR = 0.995, 95% CI: 0.992–0.997). The full results of the logistic regressions are shown in Table 3.

A summary of the results per type of factor is also offered below: 

Suicidality factors: no statistically significant association was found between early suicidal ideation and later suicide attempts. Early suicidal ideation was a risk factor for later suicidal ideation (OR = 2.038, 95% CI: 1.346–3.080) and later NSSI (OR = 2.455, 95% CI: 1.657–3.636). Early NSSI was associated with later NSSI (OR = 3.957, 95% CI: 3.244–4.826), suicidal ideation (OR = 2.337, 95% CI: 1.849–2.952), and suicide attempts (OR = 2.686, 95% CI: 1.770–4.074).

Sociodemographic factors: suicidal ideation (OR = 0.689, 95% CI: 0.655–0.723) and suicide attempts (OR = 0.502, 95% CI: 0.458–0.550) were inversely associated with female gender, while there was no association between gender and later NSSI.

Adverse childhood experiences: there was a statistically significant association between history of sexual abuse and the three SITB. A greater effect size was found for later NSSI (OR = 3.336, 95% CI: 2.059–5.407) and suicide attempts (OR = 3.958, 95% CI: 1.766–8.872) than for SI (OR = 1.870, 95% CI: 1.030–3.388).

Mental health symptoms: behavioural problems were a risk factor for all SITB, with an OR ranging from 1.570 (95% CI: 1.442–1.705) for suicidal ideation to 1.807 (95% CI: 1.651–1.978) for later NSSI. There was a statistically significant association between mood symptoms and later NSSI (OR = 1.341, 95% CI: 1.102–1.632) and suicidal ideation (OR = 1.370, 95% CI: 1.052–1.533) but not suicide attempts. Psychotic symptoms were also associated with later NSSI (OR = 1.846, 95% CI: 1.170–2.911) and suicidal ideation (OR = 1.905, 95% CI: 1.240–2.926) but not suicide attempts.

Service-utilisation-related factors: GP visits were inversely associated with later NSSI (OR = 0.998, 95% CI: 0.997–0.999) and suicide attempts (OR = 0.995, 95% CI: 0.992–0.997).

## 4. Discussion

In this study we explored early (age 8–14) risk factors for presenting with NSSI, suicidal ideation, and suicide attempt at the age of 18–24. Many of the factors explored had a statistically significant association with SITB.

### 4.1. Suicidality Factors

Most suicide explanatory models understand suicide behaviour as a continuum, in which suicide ideation is a necessary previous step for suicidal behaviour [26]. In our study, we did not find a statistically significant association between early suicidal ideation presenting in primary care and later suicide attempts. The long timespan between one risk factor and the studied outcome, together with the nonspecific nature of suicidal ideation, may explain this lack of association. Suicidal ideation has been found to be a fleeting, nonstable variable [27,28], which could, therefore, be understood more as a state variable than as a trait variable. Some authors argue that there are fundamental differences, including biological factors, between suicide ideators and suicide attempters and warn that most people with suicidal ideation will never attempt suicide [29]. Only about 18% of people who experience suicidal ideation will attempt suicide [4]. In our study, we found some overlap between suicidal ideation and suicide attempts as outcomes, in that 15.7% of people who attempted suicide had at least one additional GP visit due to suicidal ideation in the same period (18–24 years old). Theoretically, all of those who attempted suicide also had suicidal ideation—at least moments before the suicide attempt—but this situation is not recorded in our study, as our variables refer to the main reason for consultation for each visit. 

NSSI is highly related to suicide, sharing some risk factors with suicidal ideation and suicide attempts and being in itself an important risk factor for death by suicide. NSSI can increase the capability for suicide by means of increasing fearlessness and decreasing pain sensitivity [30]. In our study, we found that NSSI at age 8–14 years increased the risk for presenting with NSSI, suicidal ideation, and suicide attempts at 18–24 years old by 2.3–4 times. Attention should be paid to early adolescents who engage in NSSI, as this behaviour carries risks at later ages. We also found a certain overlap between NSSI and suicidal ideation, and NSSI and suicide attempts as outcomes at 18–24 years of age. 

Some authors advocate for making nuanced distinctions between different SITB, as they may have distinct associated biological markers [31,32]. We found some specificity in risk factors; for instance, early suicidal ideation, psychotic symptoms, and mood symptoms were risk factors for later suicidal ideation and NSSI, while they showed no association with later suicide attempts. Similarly, suicidal ideation and suicide attempts were more frequent among males, while there was no association between NSSI and gender. In contrast, other factors were shared for the three types of SITB, namely, early sexual abuse, behavioural problems, and early NSSI. 

### 4.2. Sociodemographic Factors

Female gender was a protective factor against suicidal ideation and suicide attempts in our study. Our finding contrasts with the previous literature that shows that nonfatal suicidal behaviour is more common among females, while death by suicide is more common in males [33]. However, our sample is composed of people presenting with mental health problems to primary care in young adulthood, which may alter the prevalence (e.g., men are more prone to seek help when they are feeling more overwhelmed compared to women asking for support earlier).

With respect to NSSI, we found no association with gender. While classically NSSI has been thought to be more prevalent among females, some studies report a similar frequency of NSSI by gender [34,35]. However, there may be gender differences regarding the age of onset (earlier for women) or the method chosen (cutting is more common among women, while self-hitting is more common among men), which our data were unable to examine [36,37]. 

### 4.3. Mental Health Symptoms

Behavioural problems were a risk factor for all SITB. Externalising symptoms can be indicative of a wide range of issues and are the way in which many children and adolescents express their distress but are often neglected in research and clinical practice relative to internalising problems. Behavioural problems at this age are associated with increased impulsivity and emotional dysregulation [38,39]. Previous studies have linked behavioural problems with NSSI and suicide attempts in adolescents [40,41].

We also found a relationship between both mood and psychotic symptoms and NSSI and suicidal ideation but not suicide attempts. Depression at an early age increases the risk of suicide and should be properly assessed [42,43]. As for psychotic symptoms, previous studies show that they are a risk factor for SITB [44], although few studies have explored the long-term consequences of early psychotic symptoms. A recent study showed that psychotic experiences in adolescence increased the risk of NSSI and suicide attempts after two years of follow-up [45]. Some mechanisms for this association are distress caused by psychotic symptoms and higher rates of depression in this population [44].

### 4.4. Life Events

We found a statistically significant association between history of sexual abuse by age 8–14 and the three SITB. This is consistent with the extensive previous literature, as sexual abuse is one of the best-known risk factors for suicidality [18,46]. A recent meta-analysis showed that the key mechanism explaining the association between sexual abuse and suicide attempts may be an increase in impulsivity because of the neurobiological changes induced by trauma [18]. Other factors must also be considered, such as depressive mood and interpersonal mistrust as a consequence of sexual abuse [46]. Of note, we found a greater effect size for NSSI and suicide attempts than for SI. Sexual abuse is a situation that poses a great risk to mental health and is often difficult to detect early. Tools that can help mitigate the risk of suicide among victims of sexual abuse include increasing access to mental healthcare and improving education and awareness, both among specialised staff and the general population [47]. We did not find an association between early changes in eating behaviour and later SITB, which may reflect the wide diagnostic significance, and thus low specificity, of eating -elated symptoms across both physical and mental health. 

### 4.5. Service-Utilisation-Related Factors

GP visits in the early years had a weak protective effect on NSSI and suicide attempts. This would point to positive preventive work by primary care providers or reflect the care that young people were receiving from parents/carers. However, the small effect size precludes drawing further conclusions. Primary care providers represent an important point of access for mental healthcare of children and adolescents. About 5% of children and adolescents have consultations with their primary care provider due to emotional symptoms [21]. They are usually the first point of contact in healthcare, are responsible for initial identification and treatment, and refer to mental health specialists. Hence, GPs are crucial gatekeepers for suicide prevention. Among young people who died by suicide, 10–36% had contacted their primary care provider in the month prior to their death for whatever reason, while 42–82% had contacted them in the year before death [22]. 

### 4.6. Strengths and Limitations

One of the strengths of our study is the large sample size and its high representativeness of the general UK population. Furthermore, our study covers a long follow-up period. Among the limitations, our data come from primary care services and not specialised mental healthcare services. However, this can also be a strength since it is not common to explore early risk factors in relevant settings where children and adolescents are seen for other health problems, such as GP surgeries, so GPs have a unique opportunity to identify those as a preventative measure before young people’s presentations deteriorate further. Additionally, we did not have access to data on mortality by suicide, so this was not an outcome we could use. We were unable to assess the impact of some demographic factors such as ethnicity or socioeconomic status due the lack of data in CPRD. Similarly, data on adverse childhood experiences other than sexual abuse were not available. Finally, we did not explore the absolute frequency of each symptom in each child. Rather, we explored the existence of a consultation that was coded by the GP for that reason. Thus, if a patient had a symptom but did not attend the GP for that reason or the GP did not record it, that symptom will not have been recorded in our database. This may explain the low frequency found for certain symptoms, such as NSSI, which have a higher prevalence in the previous literature in other settings, such as mental health services.

## 5. Conclusions

We identified several early risk factors for presenting SITB in young adulthood. Some of the early risk factors identified are early NSSI, early suicidal ideation, mood and psychotic symptoms, reported sexual abuse, and behavioural symptoms. The results of our study indicate that early symptoms in late childhood/early adolescence can be the start of long-standing problems going into adult life. Greater investment/support is needed for this group to improve long-term outcomes in those at risk of SITB in early adulthood. The training of primary care providers in suicide risk assessment as well as proper co-ordination with child and adolescent mental health services is crucial for suicide prevention.

## Figures and Tables

**Table 1:** Frequency of self-injurious thoughts and behaviours at 18–24 years of age (proportion of the participants who had the specified event at least once, when aged 18–24).

Self-Injurious Thoughts and Behaviours	N (%)
Any	14,288 (6.51%)
Non-suicidal self-injury	6204 (2.83%)
Suicidal ideation	7499 (3.42%)
Suicide attempt	1964 (0.89%)
Non-suicidal self-injury + Suicide ideation	970 (0.44%)
Non-suicidal self-injury + Suicide attempt	215 (0.10%)
Suicidal ideation + Suicide attempt	264 (0.12%)
Non-suicidal self-injury + Suicide ideation + Suicide attempt	70 (0.03%)

**Table 2:** Description of the sample at age 8–14 (total n = 219,581) and prevalence of symptoms/behaviours.

	Self-Injurious Thoughts and Behaviours at 18–24 Years Old (n = 14,288)	No Self-Injurious Thoughts and Behaviours at 18–24 Years Old (n = 205,293)
	N (%)	N (%)
Gender		
Male	6321 (44.2%)	75,162 (36.61%)
Female	7962 (55.73%)	130,115 (63.38%)
Non-suicidal self-injury	240 (1.68%)	794 (0.39%)
Suicidal ideation	63 (0.44%)	234 (0.11%)
Mood symptoms	242 (1.69%)	2401 (1.17%)
Anxiety symptoms	134 (0.94%)	1514 (0.74%)
Psychotic symptoms	40 (0.28%)	271 (0.13%)
Behavioural problems	1356 (9.46%)	11,004 (5.36%)
Eating problems	25 (0.17%)	281 (0.14%)
History of sexual abuse	31 (0.22%)	164 (0.08%)

**Table 3:** Early (age 8–14) risk factors for attempting suicide, experiencing suicidal ideation, and non-suicidal self-injury at age 18–24: results of the adjusted logistic regression.

	OR	95% CI		*p* Value
		Lower Bound	Upper Bound	
Risk factors for NSSI				
Gender (female)	1.032	0.978	1.088	0.254
Non-suicidal self-injury	3.957 *	3.244	4.826	<0.001
Suicidal ideation	2.455 *	1.657	3.636	<0.001
History of sexual abuse	3.336 *	2.059	5.407	<0.001
Behavioural problems	1.807 *	1.651	1.978	<0.001
Eating problems	1.191	0.645	2.199	0.576
Mood symptoms	1.341 *	1.102	1.632	0.003
Anxiety symptoms	1.114	0.850	1.461	0.434
Psychotic symptoms	1.846 *	1.170	2.911	0.008
Frequency of primary care visits	0.998	0.997	0.999	0.004
Age at first primary care visit	0.990	0.976	1.004	0.188
Risk factors for suicidal ideation				
Gender (female)	0.689 *	0.655	0.723	<0.001
Early Non-suicidal self-injury	2.337 *	1.849	2.952	<0.001
Early Suicidal ideation	2.038 *	1.346	3.080	0.001
History of sexual abuse	1.870 *	1.030	3.388	0.039
Behavioural problems	1.570 *	1.442	1.705	<0.001
Eating problems	0.750	0.380	1.478	0.405
Mood symptoms	1.370 *	1.052	1.533	0.013
Anxiety symptoms	1.095	0.851	1.407	0.481
Psychotic symptoms	1.905 *	1.240	2.926	0.003
Frequency of primary care visits	1.000	0.999	1.001	0.939
Age at first primary care visit	0.971 *	0.960	0.985	<0.001
Risk factors for suicide attempt				
Gender (female)	0.502 *	0.458	0.550	<0.001
Non-suicidal self-injury	2.686 *	1.77	4.074	<0.001
Suicidal ideation	1.685	0.717	3.958	0.231
History of sexual abuse	3.958 *	1.766	8.872	0.001
Behavioural problems	1.586 *	1.360	1.849	<0.001
Eating problems	1.262	0.400	3.978	0.691
Mood symptoms	1.125	0.758	1.671	0.558
Anxiety symptoms	1.055	0.631	1.762	0.839
Psychotic symptoms	1.334	0.504	3.529	0.562
Frequency of primary care visits	0.995	0.992	0.997	<0.001
Age at first primary care visit	1.039 *	1.014	1.065	0.002

* Statistically significant at *p* < 0.05. All results were controlled by primary care health centre.

## Data Availability

Data are contained within the article.
